# Human Cord Blood-Derived AC133+ Progenitor Cells Preserve Endothelial Progenitor Characteristics after Long Term *In Vitro* Expansion

**DOI:** 10.1371/journal.pone.0009173

**Published:** 2010-02-11

**Authors:** Branislava Janic, Austin M. Guo, A. S. M. Iskander, Nadimpalli Ravi S. Varma, Alfonso G. Scicli, Ali S. Arbab

**Affiliations:** 1 Cellular and Molecular Imaging Laboratory, Department of Radiology, Henry Ford Hospital, Detroit, Michigan, United States of America; 2 Department of Women's Health Services, Gynecologic Oncology, Henry Ford Hospital, Detroit, Michigan, United States of America; 3 Department of Eye Care Services Research, Henry Ford Hospital, Detroit, Michigan, United States of America; City of Hope National Medical Center, United States of America

## Abstract

**Background:**

Stem cells/progenitors are central to the development of cell therapy approaches for vascular ischemic diseases. The crucial step in rescuing tissues from ischemia is improvement of vascularization that can be achieved by promoting neovascularization. Endothelial progenitor cells (EPCs) are the best candidates for developing such an approach due to their ability to self-renew, circulate and differentiate into mature endothelial cells (ECs). Studies showed that intravenously administered progenitors isolated from bone marrow, peripheral or cord blood home to ischemic sites. However, the successful clinical application of such transplantation therapy is limited by low quantities of EPCs that can be generated from patients. Hence, the ability to amplify the numbers of autologous EPCs by long term *in vitro* expansion while preserving their angiogenic potential is critically important for developing EPC based therapies. Therefore, the objective of this study was to evaluate the capacity of cord blood (CB)-derived AC133+ cells to differentiate, *in vitro*, towards functional, mature endothelial cells (ECs) after long term *in vitro* expansion.

**Methodology:**

We systematically characterized the properties of CB AC133+ cells over the 30 days of *in vitro* expansion. During 30 days of culturing, CB AC133+ cells exhibited significant growth potential that was manifested as 148-fold increase in cell numbers. Flow cytometry and immunocytochemistry demonstrated that CB AC133+ cells' expression of endothelial progenitor markers was not affected by long term *in vitro* culturing. After culturing under EC differentiation conditions, cells exhibited high expression of mature ECs markers, such as CD31, VEGFR-2 and von Willebrand factor, as well as the morphological changes indicative of differentiation towards mature ECs. In addition, throughout the 30 day culture cells preserved their functional capacity that was demonstrated by high uptake of DiI fluorescently conjugated-acetylated-low density lipoprotein (DiI-Ac-LDL), *in vitro* and *in vivo* migration towards chemotactic stimuli and *in vitro* tube formation.

**Conclusions:**

These studies demonstrate that primary CB AC133+ culture contained mainly EPCs and that long term *in vitro* conditions facilitated the maintenance of these cells in the state of commitment towards endothelial lineage.

## Introduction

Over the last few decades stem cells/progenitors became focus of the development of cell therapy approaches for various diseases, including vascular ischemic diseases. The crucial step in rescuing tissues from ischemia is improvement of vascularization that can be achieved by promoting neovascularization or growth of new blood vessels. Formation of blood vessels involves two processes: vasculogenesis and angiogenesis [1]. Vasculogenesis was believed to be restricted to embryonic development and is achieved by *in situ* differentiation of the primitive progenitors, i.e. angioblasts into mature endothelial cells (ECs) and their organization into a primary capillary network [2], [3]. In contrast, angiogenesis occurs both during embryonic development and postnatal life and is carried out by endothelial migration and sprouting from preexisting vessels [4], [5]. For a long time it was considered that postnatal blood vessel formation is restricted to angiogenesis only. Since numerous previous studies demonstrated the existence of circulating ECs in peripheral blood in various vascular diseases [6], [7], [8], [9], [10] the question was asked whether these cells (or their precursors) play a role in the postnatal vascular growth. The ground breaking evidence implicating the possibility that endothelial stem or precursor cells may contribute to the formation of new blood vessels in adults, by vasculogenesis, came from the work by Asahara et al. [11]. They demonstrated the presence of CD34+ endothelial progenitors in human peripheral blood, capable of incorporating into the sites of active angiogenesis. Since then, researchers have gained significant insights into the postnatal vascularization, and endothelial progenitor cells (EPCs) in particular, have been suggested to take part in these processes by homing to the sites of neovascularization and by differentiating into ECs. Therefore, EPCs seem like excellent candidates for developing therapeutic approaches for human vascular diseases.

In spite of the growing evidence on therapeutic potential of EPCs, definition, phenotypic and functional characterization as well as angiogenic role of the putative EPC still remains controversial. EPCs are considered as bone marrow-derived cells that have the capacity to proliferate, circulate and differentiate into mature ECs. However, many groups used adult peripheral blood and placental cord blood as a source for endothelial progenitors [12], [13], [14], with a various degree of success in demonstrating angiogenic properties of these differently isolated starting population. In addition, EPCs propagated under different culture conditions, such as suspension vs. adherent culture, and in the presence of different cytokine combinations, exhibited significant differences with regard to phenotypes, function and very often, proliferative potential as well [14], [15]. Earlier studies defined EPCs as the cells co-expressing hematopoietic stem cell (HSC) marker CD34 and EC marker, vascular endothelial growth factor receptor 2 (VEGFR2) [16]. However, subsequent studies demonstrated that some mature ECs also co-express CD34 and VEGFR2. In addition, CD34 was shown not to be an exclusive marker for hematopoietic cells and novel AC133 glycoprotein was accepted as a more suitable marker for immature progenitor cells [14], [17] that is expressed on HSC but not on mature ECs [18], [19]. A subset of circulating CD34+ cells that express both, VEGFR-2 and AC133, was also suggested to represent a functional EPC population that plays a role in postnatal angiogenesis or vasculogenesis [17]. It has been hypothesized that CD133+/CD34+/VEGFR2+ cells represent an immature, highly proliferative EPC population that gives rise to CD133-/CD34+/VEGFR2+ cells that are more mature and limited in their proliferative capacity [20]. Through the process of differentiation into mature ECs, EPCs down-regulate the expression of CD34 and AC133 [19], [21] and continuous culturing increases the expression of mature EC markers [22]. However, the overlap of phenotypical and functional properties among EPCs, HSCs and ECs remains the source of controversy and it is still not clear what the “real” identity of EPCs is and whether their angiogenic potential is contingent on differentiation/maturation status. Nevertheless, the majority of studies using progenitors from bone marrow, peripheral or cord blood demonstrated the ability of these cells to self-renew, circulate and differentiate and these properties are central to the development of EPC-based therapies. However, the successful clinical application of such transplantation approach is limited by low quantities of EPCs that can be generated form a patient. Hence, the ability to *in vitro* amplify the numbers of autologous EPCs while preserving their angiogenic potential, is critically important for developing EPC based therapies for ischemic vascular diseases.

The aim of this study was to establish the optimal conditions for the *in vitro* expansion of cord blood (CB) derived AC133+ progenitor cells that would enable long term expansion of cell numbers without affecting angiogenic potential of the expanded population. We analyzed the phenotypical and functional characteristics of CB AC133+ cells that were expanded *in vitro* in the presence of SCF, TPO and Flt3 for up to 30 days. Our results indicated that long term *in vitro* culturing did not impede the CB AC133+ potential to give rise to the population exhibiting endothelial cell type characteristics.

## Materials and Methods

### Ethics Statement

The use of human cord blood in this study was approved by a Henry Ford Health System Institutional Review Board (IRB). Written informed consent was obtained and the consent process was maintained under IRB-approved security protocol using an IRB-approved consent form and the process of consent. Animal experiments were performed according to the protocol approved by our animal care and user committee at Henry Ford Health System.

### Isolation and *In Vitro* Culture of AC133+ EPC

Progenitor cells positive for CD133+ marker (AC133+) were isolated from the cord blood obtained from volunteers entered in Institutional Review Board (IRB) approved protocols. The cord blood mononuclear cell population was generated by Ficoll gradient centrifugation and was enriched for AC133+ cells by immunomagnetic positive selection using the MidiMACS system (Miltenyi Biotec, Auburn CA) according to the manufacturer's protocol. Freshly isolated AC133+ cells were suspended in Stemline II media (Sigma, St. Louis, MO) supplemented with 40 ng/ml of stem cell factor (SCF), 40 ng/ml of FLT3 and 10 ng/ml of thrombopoietin (TPO) (all from CellGenix, IL). Cells were maintained under these conditions as a suspension culture for 30 days with the cell concentration kept between 5×10^5^–1×10^6^ cells/ml. Throughout the 30 days of long term expansion, cells were cultured in BD Falcon™ 6-well cell culture dishes (BD Biosciences) and monitored daily by inverted phase contrast microscopy. At splitting, the number of live cells was determined by Trypan blue exclusion assay and the data was used to generate growth curve. Upon determining the cell count, cells were split by adding freshly prepared media to adjust the concentration to 5×10^5^ cells/ml.

### Human Dermal Microvascular Endothelial Cells (HDMVEC)

Human dermal microvascular endothelial cells (HDMVEC) were purchased from Cambrex (East Rutherford, NJ). Cells were grown in microvessel growth supplement media 131 (Cambrex East Rutherford, NJ) according to the manufacturer's recommendations and maintained at 37°C in a humidified incubator containing 5% CO_2_.

### *In Vitro* Differentiation of AC 133+ EPCs

Differentiation potential of primary long term AC133+EPC culture was assessed at days 5–6, 10–15 and 25–30 of *in vitro* expansion. Cells were suspended in Stemline II media supplemented with 2% FBS and 2 ng/ml of Vascular Endothelial Growth Factor (VEGF) and plated in chamber slides coated with fibronectin at a concentration of 2×10^5^/ml. Cells were allowed to differentiate for 2 weeks. Every 2–3 days, old media was replenished with fresh media, in each chamber, and cells were monitored by inverted phase contrast microscopy to assess the morphological changes associated with differentiation. After 2 weeks of differentiation cell were analyzed by flow cytometry and immunocytochemistry for the expression of EPC differentiation markers.

### Flow Cytometry

Cells expanded as suspension culture under the growth conditions or cells grown under the differentiation conditions were harvested, washed in ice cold 1x PBS and incubated for 30 min on ice, in dark, with the respective fluorescently labeled antibodies. Fluorescence activated flow cytometry was performed with LSR II (Becton Dickinson) flow cytometer and a minimum of 10,000 events were analyzed for each sample. Live cells used for the analysis, were gated based on forward angle light scatter (FSC) and side angle light scatter (SSC) characteristics and further analyzed using the Cell Quest Pro software (Becton Dickinson). Specific antibodies that were used in flow cytometric experiments to analyze the expression of cell surface markers were: mouse anti-human CD133 IgG1 (Miltenyi Biotec, Auburn CA), mouse anti-human CD34 IgG1 (BioLegend), mouse anti-human CD45 IgG1 (BioLegend), mouse anti-human CD117 IgG1 (BioLegend), mouse anti-human CD14 IgG1 (BioLegend), mouse anti-human CD20 IgG2b (BioLegend), mouse anti-human CD3 IgG2b (BioLegend), mouse anti-human CD29 IgG1 (BioLegend), mouse anti-human CD31 IgG1 (BioLegend), mouse anti-human CD54 IgG2a (BioLegend), mouse anti-human KDR (VEGFR2) IgG1 (R&D Systems), mouse anti-human VE-Cadherin IgG2b (R&D Systems), mouse anti-human CD106 IgG1 (BioLegend), mouse anti-human CD62E IgG2a (BioLegend), mouse anti-human CD184 IgG2a (BioLegend), mouse anti-human CD195 IgG2a (BioLegend), mouse anti-human CD105 IgG2a (BioLegend), mouse anti-human CD150 IgG1, mouse anti-human CD140 A and B IgG1 (BioLegend). All the antibodies were used in the concentrations suggested by the suppliers.

### Immunocytochemistry

Differentiated AC133+ EPCs were analyzed by immunocytochemistry for the expression of endothelial cell specific markers. The following specific antibodies were used: mouse anti-human anti CD31 (DakoCytomation), rabbit anti-human anti CD309 (VEGFR2 or KDR) (Thermo Scientifics) and rabbit anti-human anti von Willebrand Factor (vWF)(DakoCytomation). Positive staining was detected using Texas Red or FITC conjugated secondary antibodies (Jackson ImmunoResearch, Inc). Negative control samples included cells treated with secondary antibodies only. All the antibodies were used in the concentrations suggested by the suppliers. To visualize nuclei, cells were counterstained with DAPI nuclear stain. Cells were analyzed by fluorescent microscopy.

### EPCs Migration in Response to SDF-1 and Rantes

Chemotactic assay was performed using QCM™ Chemotaxis 3 µm pores 96-well Cell Migration Assay kit (Chemicon) according to the supplier's experimental protocol. In brief, 1×10^5^ cells were suspended in 100 µl of serum free media and placed in the upper compartment of transwell chamber. Lower compartments were filled with 150 µl of serum free media, with or without 50 ng/ml of SDF-1 (CXCL12) or 50 ng/ml of Rantes (CCL5). Each sample was assayed in triplicates and after incubation of 4 h at 37°C, cell migration was detected as fluorescence that was read using 520 nm filter set. EPCs migration in response to Rantes and SDF-1 was compared to the migration observed in control EPCs incubated without chemoatractant and EPCs incubated with 10% FBS (positive control).

### Endothelial Tube Formation in Matrigel

Ability of CB AC133+ cells to differentiate and organize into capillary-like structures in the presence of basement membrane matrix was analyzed using BioCoat Matrigel 35 mm Cellware (BD Bioscience) according to the manufacturer's suggestions. To assess the possible paracrine proangiogenic effect of CB AC133+ cells on microvascular endothelial cells, HDMVEC were cultured on BioCoat Matrigel together with CB AC133+ cells or in the presence of CB AC133+ cells' conditioned media (supernatant collected from CB AC133+ cell culture). In brief, 2×10^4^ of EPCs were plated onto the rehydrated Matrigel in the media free of serum and growth factors and incubated for 24 hours at 37°C, 5% CO_2_. Tube formation was monitored by microscope. For the co-culture experiments, CB AC133+ cells were labeled with red fluorescent dye DiI (Molecular Probes) while HDMVEC were labeled with green fluorescent reagent Calcein (Molecular Probes). Labeling procedures were done following the manufacturers' protocols. CB AC133+ cells (2×10^4^) were mixed with HDMVEC (1×10^5^). In one set of wells HDMVEC (1×10^5^) were incubated in the presence of CB AC133+ cells' supernatants only. Tube formation was assessed according to the manufacture's recommendations.

### Incorporation of DiI-Ac-LDL

At days 5–6, 10–15 and 25–30 of *in vitro* primary culture cells were differentiated for 2 weeks as described. Upon differentiation, cells were incubated in the presence of 10 mg/ml of acetylated low density lipoprotein, labeled with 1,1′-dioctadecyl – 3,3,3′,3′-tetramethyl-indocarbocyanine perchlorate, DiI-Ac-LDL (Biomedical Technologies, Inc). After 4 h of incubation at 37°C, 5%CO_2,_ cells were washed with probe free-media, fixed in 3% paraformaldehyde and analyzed by fluorescent microscopy using rhodamine excitation/emission filters.

### Preparation of Ferumoxides-Protamine Sulfate (FePro) Complex and Labeling of CB AC133+ Cells

For the purpose of labeling, CB AC133+ cells were suspended at the concentration of 4×10^6^ cell/ml in serum free RPMI and plated in 24-well plate cell culture dish, 0.5 ml per well. The labeling procedure was performed according to our previously published method [23]. In brief, commercially available ferumoxides suspension (Fe) (Feridex IV; Bayer-Schering Pharma, Wayne, NJ, USA) was first added to the cells by directly pipetting into the culture dish to the final concentration of 100 µg/ml. Immediately after, preservative-free protamine sulfate (Pro) (American Pharmaceuticals Partners, Shaumburg, IL, USA) was added in the same manner to the final concentration of 3 µg/ml. Pro was supplied as 10 mg/ml of stock solution and was freshly diluted to a concentration of 1 mg/ml in distilled water at the time of use. Cells were incubated in the presence of FePro complexes for 15 minutes at 37°C, 5% CO2, after which complete growth media was added (0.5 ml per well) and the labeling procedure was further continued for 4 h at 37°C, 5% CO_2_. Upon labeling, cells were harvested, washed two times with 1x PBS and 3×10^6^ cells suspended in 100 µl of 1x PBS were intravenously administered to matrigel implant bearing Balb/c nude mice. Cell labeling efficiency was determined by Prussian blue staining and by determining the intracellular iron concentration according to our published method [23].

### Subcutaneous Matrigel Plug

Animal experiments were performed according to the protocol approved by our animal care and user committee at Henry Ford Health System. Subcutaneous matrigel plug mouse model was generated using 6 to 8 weeks old female or male Balb/c nude mice (Charles River Laboratories, MD). To mimic angiogenic environment of the ischemic tissue that would attract circulatory EPCs, 300 µl of Matrigel (BD Bioscience) was mixed with SDF-1 chemoatractant (50 ng/ml) and subcutaneously implanted into the mice flanks. Immediately after implantation mice received 3×10^6^ of FePro labeled EPCs via I.V. injection after which angiogenesis within the matrigel was allowed to develop for 7 days. Mice were euthanized 7 days after matrigel implantation, matrigel plugs with the surrounding tissues were harvested and analyzed by *ex vivo* MRI and immunohistochemistry.

### *Ex Vivo* MR Imaging Protocol

The mice were deeply sedated using CO_2_ inhalation and euthanized. Immediately after, animals were perfused through the left ventricle with PBS followed by 4% paraformaldehyde (PFA) after which matrigel implants with surrounding tissues were removed and placed into 4% PFA and 3% sucrose. *Ex vivo* MRI was performed using a 3 Tesla clinical MRI (GE Excite) with a dedicated small animal-imaging coil (Dody scientific, SC). Fixed tissues were imaged in fomblin perfluoropolyether (Fomblin, Ausimont USA, Thorofare, NJ). Three-dimensional 200 µm isotropic 3D FIESTA images were obtained using TR = 17.35 ms, TE = 8.58 ms, FA = 40, 6×6 cm FOV, 300×300 matrices, 0.2 mm thick, NEX = 2. Multiplanar reconstruction was performed using Voxar 3D software.

### Immunohistochemistry and Prussian Blue Staining

After the *ex-vivo* MR imaging, matrigel plug with surrounding tissues were prepared for frozen sectioning. Ten micron sections were cut and stained with FITC conjugated tomato lectin to visualize the newly formed vasculature while nuclei were visualized with DAPI. The very adjacent tissue sections were stained with DAB enhanced Prussian blue to confirm the presence of FePro labeled administered CB AC133+ cells. Prussian blue staining was performed according to our previously reported method [24].

### Statistical Analysis

Each experiment was performed at least two times and each sample was tested in triplicate. Data are expressed as mean±SD. Statistically significant difference was determined with one way ANOVA analysis followed by Fisher's PLSD post-hoc test, when there were more than two groups. For analysis between two groups student-t test was used. A value of p<0.05 was considered significant.

## Results

### Growth Potential and Endothelial Progenitor Characteristics of CB AC133+ Cells during Long Term *In Vitro* Culture

**Figure 1:**
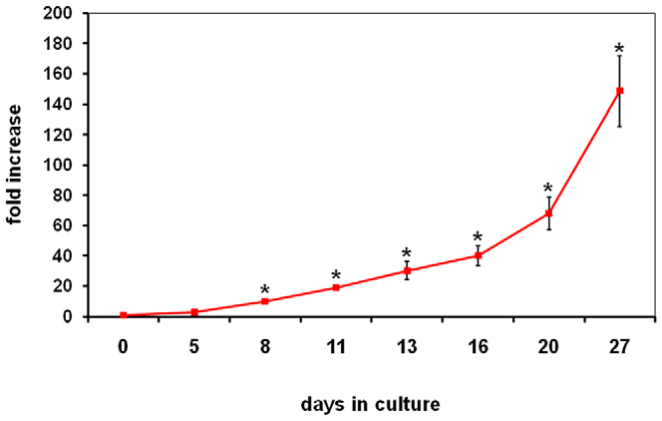
Growth kinetics of CB AC133+ cells. CB AC133+ cells were cultured in the presence of 40 ng/ml of stem cell factor (SCF), 40 ng/ml of FLT3 and 10 ng/ml of thrombopoietin (TPO) for 30 days. Cell numbers were determined by Trypan blue exclusion assay at the time points indicated on the graph. During the ‘lag phase’ (day 0–8) there was no significant increase in numbers with time. Starting at day 8 the living cell population increased rapidly with time at an exponential growth in numbers, and the growth rate increasing with time. Increase in the cell numbers expressed as fold increase (cell numbers at splitting/cell numbers at day 0). Data points, means ± SD. * p<0.05.

**Figure 2:**
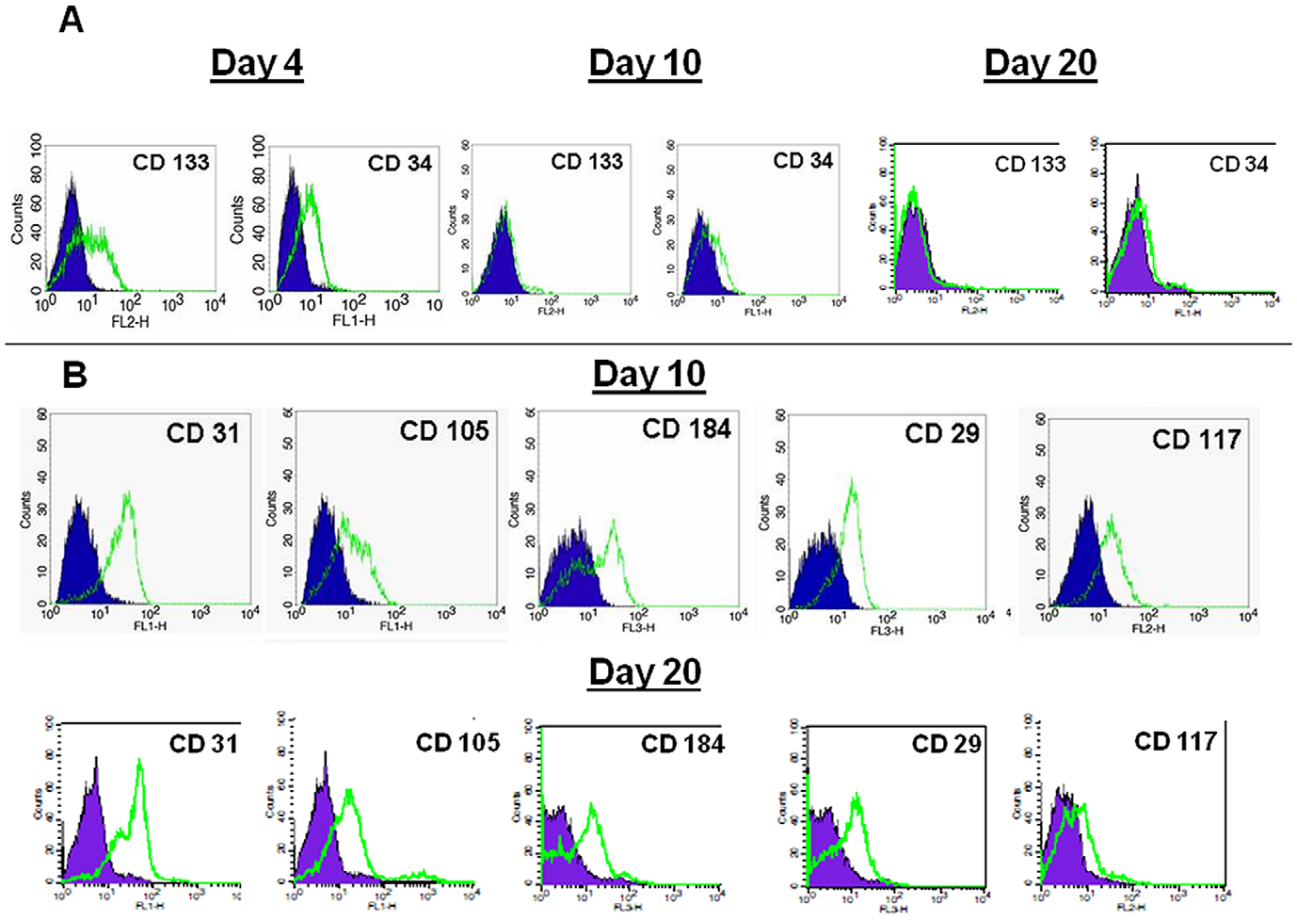
Expression of cell surface markers in CB AC133+ progenitor cells during long term *in vitro* culture. The data depicts the levels of CD133 and CD34 protein expression in AC133+ cells cultured for 4, 10 and 20 days (**A**) and the levels of CD31, CD105, CD184, CD29 and CD117 in AC133+ cells cultured for 10 and 20 days (**B**). Flow cytometric histograms from one representative experiment are shown (n = 3). At least 10,000 live gated cells were analyzed for FITC, PE or PE-Cy5 expression. Isotype controls are shown as solid blue histograms.

Although the majority of previous work on isolation and characterization of the putative EPC utilized adherent culture conditions as a mode for *in vitro* cell propagation, we investigated the possibility of using suspension culture for CB AC133+ cells long term expansion. Thus, during the 30-day culture we analyzed the growth potential of CB AC133+ and the expression of cell surface markers indicative of endothelial progenitor phenotype. Upon isolation from cord blood, AC133+ cells were grown in media containing 40 ng/ml of stem cell factor (SCF), 40 ng/ml of Fms-related tyrosine kinase 3 ligand (Flt3 ligand) and 10 ng/ml of thrombopoietin (TPO). We started the long-term expansion with 5.8×10^5^–6.8×10^5^ cells per culture. Cells were maintained in culture for 30 days at the concentration of 5×10^5^–1×10^6^ cells/ml and the fold expansion was determined by periodically counting the numbers of live cells using the Trypan blue exclusion assay. As shown in Figure 1, significant increase in cell numbers was observed after 8 days of cell expansion. This significant difference in cell numbers, compared to the cell numbers at day 0, was maintained during 4 weeks of culturing and at day 27 of culture cells achieved 148 fold expansion (**Figure 1**). As markers for immature endothelial progenitors, we examined AC133 and CD34. Upon isolation, majority of cells exhibited the expression of AC133 and CD 34 (>90%) (data not shown). As expected, at day 4, cells down-regulated the expression of AC133 and CD34 with the complete loss of AC133 expression by day 10 and CD34 by day 20 of primary culture (**Figure 2A**). In addition, by day 10 in culture, cells upregulated the expression of EC surface markers such as CD31, CD105, CD184 and CD29, and as expected, this increase in expression was maintained at the later time (day 20) in culture (**Figure 2B**). The expression of CD117 (c-kit) marker was still present on more than 50% of cells at day 10 and on 15% of the cells at day 20, indicating the presence of actively proliferating progenitors.

### The Effect of Long Term *In Vitro* Expansion on Differentiation Potential of CB AC133+ Cells

**Figure 3:**
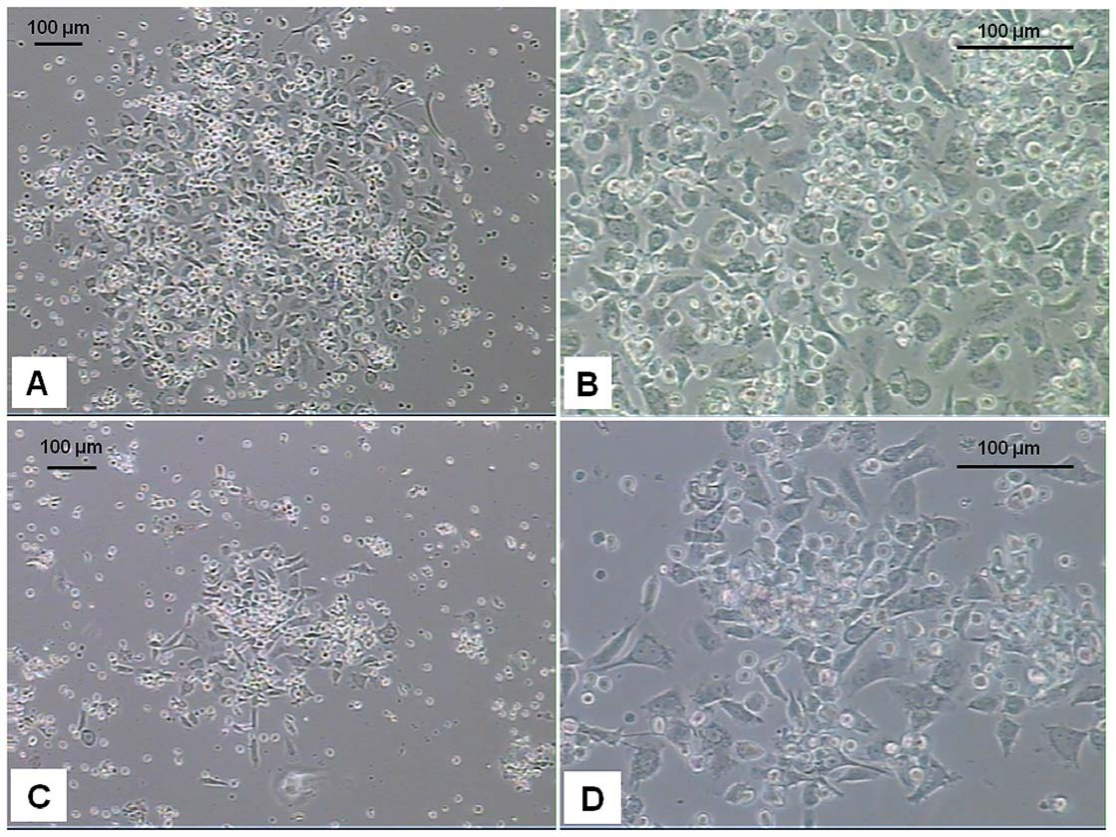
Morphological changes in CB AC133+ cells differentiated for 2 weeks. The similar pattern of morphological changes was observed in cells differentiated at day 15 (**A, B**) and at day 30 (**C, D**) of primary culture. Differentiated cells exhibited “cobble stone”-like organizational pattern. Spindle shaped adherent cells sprouted from cell clusters in an attempt to organize into linear, tube-like structures. Phase contrast photomicrographs from 2 representative experiments. Magnifications used: 10x (A and C) and 25x (B and D). Scale bar  = 100 µm.

**Figure 4:**
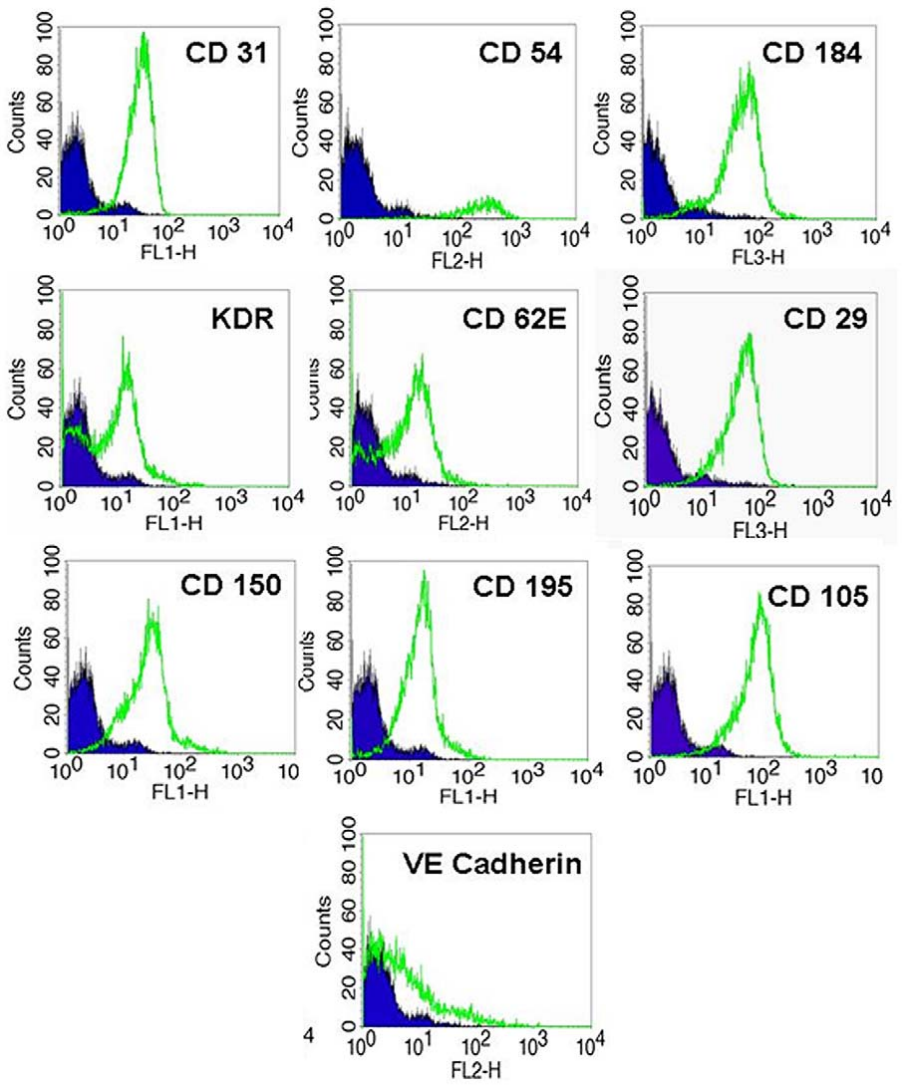
Expression of cell surface markers in CB AC133+ progenitor cells differentiated for two weeks. CB AC133+ progenitor cells at day 31 of primary culture were differentiated for two weeks. Flow cytometric analysis showed that after 2 weeks of differentiation cells were positive for CD31, CD54, CD184, KDR, CD62E, CD29, CD150, CD195, CD105 and VE Cadherin. Flow cytometric histograms from one representative experiment are shown (n = 3). At least 10,000 live gated cells were analyzed for FITC, PE or PE-Cy5 expression. Isotype controls are shown as solid blue histograms.

**Figure 5:**
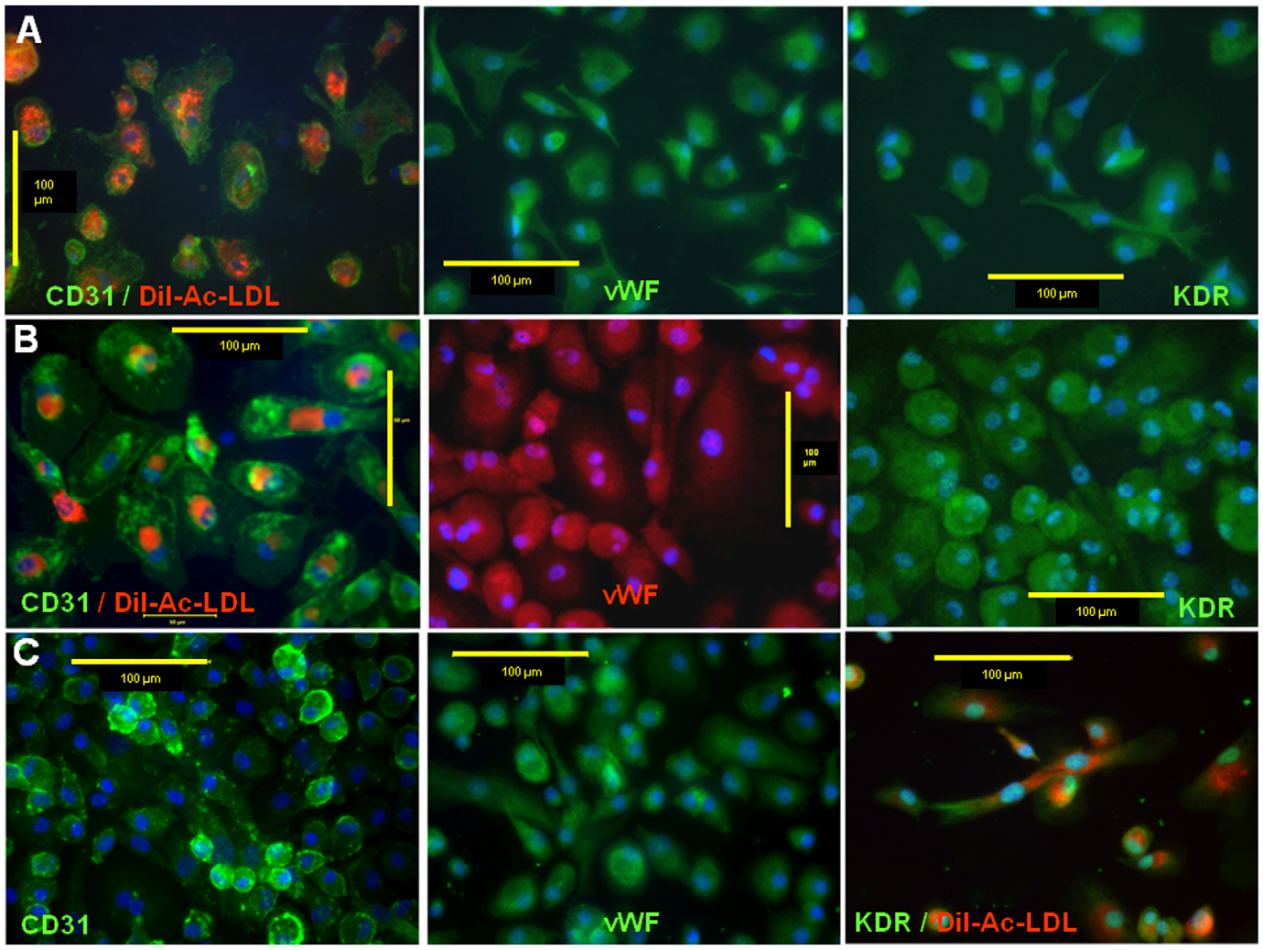
Expression of CD31, vWF and KDR in differentiated CB AC133+ progenitors. Cells were induced to differentiate at day 5–6 (**A**), day 10–15 (**B**) and day 25–30 (**C**) of primary culture. Positive signals were visualized with FITC conjugated secondary antibody (green) and Rho conjugated secondary antibody (red). Nuclei were visualized with DAPI (blue). CD31 positive (left panels in A and B) and KDR positive (right panel in C) cells also exhibited the uptake of DiI-Ac-LDL (red). Photomicrographs (40x) of differentiated cells representative of 3 other experiments. Scale bar  = 100 µm.

To characterize the differentiation potential of long term CB AC133+ culture to give rise to EC type, cells were plated on fibronectin coated surface in the presence of 2% FBS and 2 ng/ml of VEGF at days 5–6, 10–15 and 25–30 of primary culture. Cells were differentiated for 2 weeks and analyzed for the changes in cell morphology and expression of mature EC specific markers. During the course of differentiation, similar patterns with regard to cell size and shape were observed in cells that were induced to differentiate at different time points post-isolation. As revealed by light microscopy, in all the experiments, at about day 10 of differentiation, majority of cells attached to the surface forming the areas exhibiting “cobble stone”-like organizational pattern, and spindle-shaped adherent cells sprouted from cell clusters in an attempt to organize into linear, tube-like structures. The similar pattern of morphological changes was observed in cells differentiated at day 10–15 (**Figure 3A-B**) and at day 25–30 (**Figure 3C-D**) of primary culture. In addition to the changes in cell morphology, differentiation process is associated with phenotypical changes that include changes in expression of specific cell surface proteins that play important roles in regulating functional competence of endothelial type cells. Flow cytometric analysis showed that after 2 weeks of differentiation majority of the cells were positive for CD31, CD54, CD184, KDR, CD62E, CD 29, CD150, CD 195 and CD 105 (**Figure 4**). Certain percentage (40%) of cells also expressed VE-Cadherin. Fluorescent microscopy revealed that the majority of cells exhibited expression of CD31, VEGFR2 and von Willebrand factor (vWF) when they were induced to differentiate at day 5–6 (**Figure 5A**), day 10–15 (**Figure 5B** **)** and 25–30 (**Figure 5C**) of primary culture. The observed pattern of expression and morphological features indicates that long term *in vitro* culturing did not impede CB AC133+ cells' potential to differentiate towards ECs.

### The Effect of Long Term *In Vitro* Expansion on Functional Properties of CB AC133+ Cells

To address the question whether CB AC133+ cells exhibited functional characteristics of physiologically competent ECs, uptake of acetylated low-density lipoprotein (Ac-LDL), ability to form tube like formation and migrate in response to chemoatractants were tested.

Acetylated – LDL is taken up by ECs via the “scavenger cell pathway” of LDL metabolism and this metabolism in ECs has been shown to be at the accelerated rate compared to other cells types [25], [26]. Uptake of Ac-LDL was very often used to identify EC population [27], [28] and to assess ECs' functional integrity [29], [30]. Therefore we analyzed the uptake of DiI-Ac-LDL in CB AC133+ cells that were differentiated for two weeks at days 5–6, 10–15 and 25–30 of primary culture. As shown in **Figure 5A** (first panel), **Figure 5B** (first panel) and **Figure 5C** (last panel) differentiated cells exhibited uptake of DiI-Ac-LDL that was displayed as uniform, perinuclear red fluorescence. This pattern is typical of mature ECs and it indicated the presence of functional ECs population.

**Figure 6:**
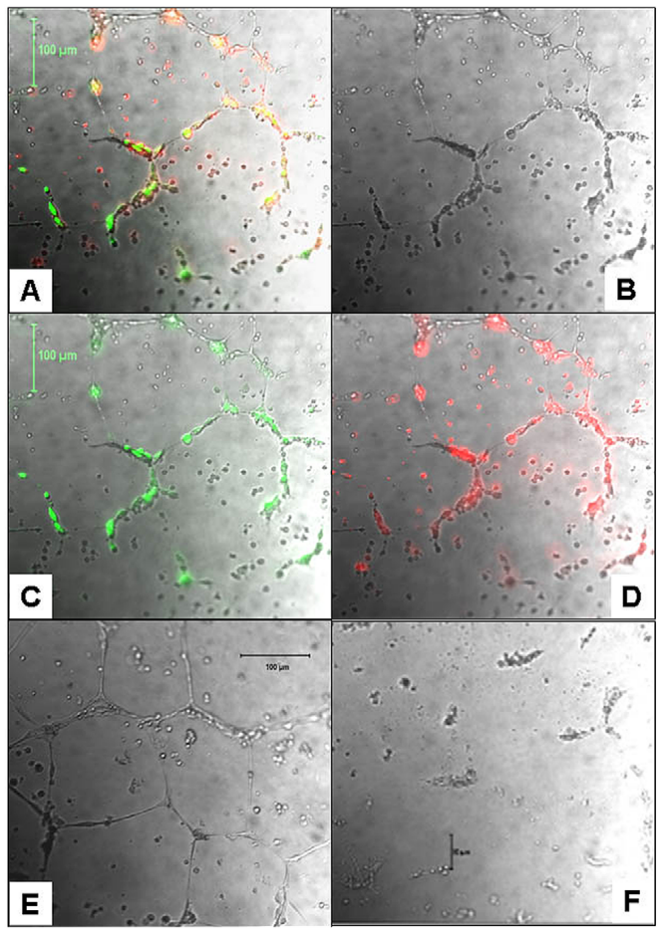
Functional analysis of long term expanded CB AC133+ cells to form tubes in Matrigel. Tube like structures after 24 h of CB AC133+ cells and HDMVECs co-culture (**A-D**). Complete tubes in matrigel formed by HDMVECs incubated in the presence of CB AC133+ cells' supernatants (w/o EPCs and VEGF) for 24 h (**E**). When plated alone, CB AC133+ did not form tube like structures (**F**). Note in panels A-D, HDMVECs labeled with Calcein (**C**, green fluorescence) and CB AC133+ cells labeled with DiI (**D**, red fluorescence) co-localized (yellow; panel **A**) to form tube like structure. Most of the green fluorescent cells appeared to be structural part of the tubes, while some of the red florescence cells that did not became part of the tube network remained scattered between the tubes. Overlays of bright light microscopy and fluorescent microscopy images (**A, C, D**). Bright light microscopy only, images shown in panels B, E and F. Magnification 10x.

The ability of long term expanded CB AC133+ cells to respond to the extracellular matrix components by forming tubular structures was analyzed at day 10–15 and 25–30 of primary culture, by plating the cells on matrigel coated dishes for 24 h. After 24 h of co-culture with HDMVEC, CB AC133+ cells exhibited migratory and organizational pattern indicative of incorporation into the tube like structures. As shown in **Figure 6A-D**, HDMVECs labeled with Calcein (green fluorescence) and CB AC133+ cells labeled with DiI (red fluorescence) co-localized to form tube like structure. Most of the green fluorescent cells appeared to be structural part of the tubes, while some of the red florescence cells that did not became part of the tube network remained scattered in between the tubes. It is plausible that structural basis for the observed tube structure came from HDMVECs, with CB AC133+ playing a supporting role. To address that question, we incubated HDMVECs in the presence of CB AC133+ cells' supernatants (w/o EPCs and VEGF) for 24 h after which HDMVECs formed complete tubes in matrigel (**Figure 6E**). This data indicated also the possible indirect, paracrine angiogenic role for CB AC133+ progenitors. When plated alone, CB AC133+ did not form tube like structures (**Figure 6F**).

**Figure 7:**
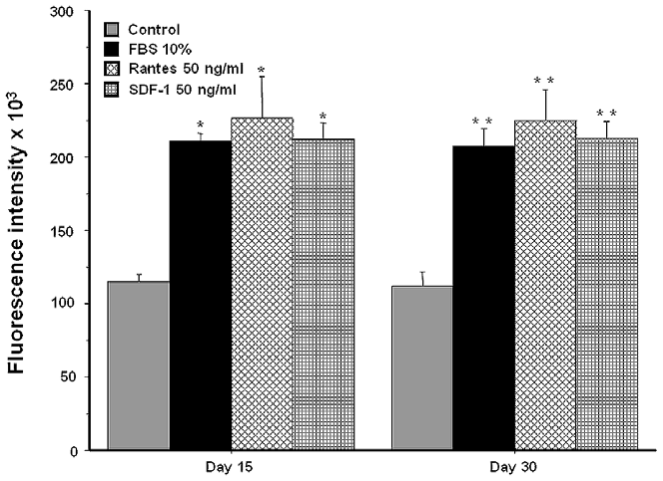
Effect of long term *in vitro* expansion on CB AC133+ cell chemotaxis in response to SDF1-α and Rantes. At days 15 and 30 of primary culture, CB AC133+ cells were incubated for 4 h in the presence of 50 ng/ml of either Rantes or SDF-1α. Cell migration in response to these to chemokines was significantly higher (p<0.05) than that observed in control conditions (w/o chemoatractant; gray bars), with no difference between the cells cultured for 15 and the cells cultured for 30 days. Cells incubated in the presence of 10% FBS (black bars) were used as a positive control. Bars, means ± SD. * p<0.05.

**Figure 8:**
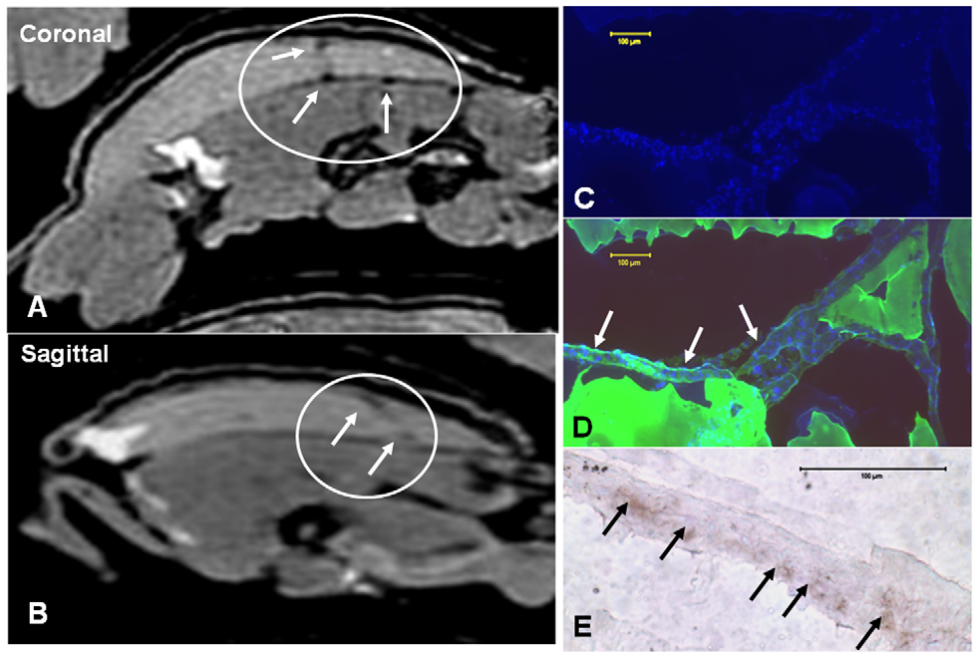
Migration and accumulation of FePro labeled CB AC133+ cells in the matrigel plug detected by MRI and immunohistochemistry. Isotropic 200 µm *ex vivo* MRI image obtained in coronal plan by FIESTA sequence (**A**) and the reconstructed 200 µm image of the sagital view (**B**). Administered FePro labeled CB AC133+ cells generated low signal intensity areas seen on both images (arrows in the circle). Fluorescent DAPI staining of the matrigel section that corresponds the MRI images show multiple cells within the matrigel (**C**) and the cells are mostly seen within a tube like structures depicted by FITC labeled tomato lectin (**D**). DAB-enhanced Prussian blue staining of the section very adjacent to the section stained with DAPI and FITC-lectin shows multiple, iron-positive, administered cells within the tube like structure (E). Scale bar  = 100 µm.

One of the important characteristics of the cells of endothelial lineage is the ability to exhibit directional migration, i.e. chemotaxis, along the concentration gradients of biologically active, low molecular weight peptides called chemokines. Therefore we analyzed the effect of long term culturing on CB AC133+ cells' chemotaxis by determining their ability to migrate along the concentration gradients of SDF-1α and Rantes chemokines under the *in vitro* conditions. In addition to *in vitro* migration, we tested CB AC133+ cells' migratory capacity *in vivo*, by employing matrigel plug model in Balb/c nude mice. At days 10–15 and 25–30 of primary culture, CB AC133+ cells were incubated for 4 h in the presence of 50 ng/ml of either SDF-1α or Rantes. Cell migration in response to these two chemokines was significantly higher (p<0.05) than that observed in control conditions (w/o chemoattractant), with no difference between the cells cultured for 10–15 and the cells cultured for 25–30 days (**Figure 7**). In addition, similar significant increase in migration was observed when cells were incubated in the presence of 10% FBS, that served a positive control for migratory response. These data indicate that during the long term culturing CB AC133+ progenitors maintained functional characteristics with regard to chemotaxis. To assess the capacity of CB AC133+ cells to *in vivo* migrate and incorporate into the sites of neovascularization we used matrigel plug model in Balb/c nude mice. After *in vitro* expansion for 10–15 days, CB AC133+ cells were magnetically labeled with FePro and administered intravenously into the matrigel implant bearing Balb/c nude mice. Our extensive previous research on a variety of cell types showed that FePro labeling does not have significant effect on cellular physiology [24], [31]. Seven days post injection MRI and immunohistochemistry revealed that FePro labeled CB AC133+ cells migrated towards neovasculature within the matrigel implants. This migration was detected by *ex vivo* MRI as hypointensity areas resulting from T2* shortening due to the presence of FePro labeled cells (**Figure 8A-B**). At the same time lectin (**Figure 8C**) and DAPI (**Figure 8D**) fluorescent, and Prussian blue (**Figure 8E**) staining of sections generated from the areas exhibiting hypointensity MRI signals revealed the co-localization of labeled cells with newly formed tube structures.

## Discussion

The hallmark of EPCs is their ability to proliferate, circulate and give rise to functional progeny. Due to these properties they have a huge therapeutic potential with respect to vessel repair and neovascularization in ischemic diseases, as well as a possible gene carriers or delivery systems in conditions such as tumors, which are closely associated with ischemia. However, isolating EPCs is a challenging task due to their very low *in viv*o numbers. Previous reports showed that bone marrow contains less than 0.05% of EPC while in the peripheral blood only 0.01% of mononuclear cells are EPCs [13], [32]. In recent years, human umbilical cord blood became an interesting source of various types of stem/progenitor cells [33], [34], [35], including hematopoietic [36], [37] and AC133+ stem/progenitor cells. Previous work have shown that the numbers of EPCs generated from cord blood are higher (up to 0.64%) than the numbers generated from human peripheral blood [13], [38]. In addition, CB AC133+ cells represent a multipotent adult stem/progenitor cell population that can self-renew and differentiate to a variety of specialized cells [38], [39], [40]. Taken together, currently available data indicate that CB AC133+ cells may be one of the best candidates for developing therapies for vascular ischemic diseases. Although the exact numbers needed for the successful *in vivo* therapeutic neovascularization are not known, *in vitro* cell amplification appears to be a necessary step in gaining the appropriate quantities of cells needed.

Having the very controversial data on the identity and source of EPCs in mind, here we ask whether cord blood derived AC133+ progenitors can preserve their endothelial cell type properties during the long term *in vitro* expansion. We showed that in the presence of SCF, Flt3 ligand and TPO isolated AC133+ population maintained endothelial precursors for at least 30 days of *in vitro* culture. The phenotypic characteristics of these precursors were exhibited as down regulation and complete loss of CD133 and CD34 marker expression early in the culture (day 4–10) and as up-regulation of CD31, CD105, CD184, CD29 and CD117. The observed down-regulation of CD133 and CD34 molecules with the increased time in culture indicated the presence of more mature endothelial progenitors. Although this phenomenon has been previously reported, the time points when significant decrease in the percentage of cells expressing CD133 and CD34 was detected ranged from day 7 to day 30 after isolation of AC133+ cells [38], [41], [42], [43]. The discrepancy in the observed dynamic of CD133 and CD34 expression, as well as in CD31, CD105, CD184, CD29 and CD117 expression may reflect the differences in culture conditions with regard to cytokine compositions and cell growth surface employed by distinct groups. Nevertheless, the overall pattern of expression at any time during the long term culture was indicative of maturation and functional status of the cultured cells. The expression of proteins such as PECAM-1 (CD31), endoglin (CD105) and integrin (CD29) indicated the commitment of these cells towards endothelial lineage. Although expressed on various other cell types, SDF-1 receptor (CD184, CXCR4) is also typically expressed on endothelial cells and is important for cellular migration within the hypoxic environment in ischemic tissues. On the other hand, at day 10 of the culture significant number of cells still maintained the expression of SCF receptor c-kit (CD117) and that expression indicated the presence of actively proliferating precursors. Although never completely disappearing, the percentage of CD117 positive cells became less at day 20 of culture. However, the CB AC133+ exhibited robust proliferative response to the culture conditions and at the very end of *in vitro* culture exhibited 148-fold population expansion. It is possible that over the course of 30 day CB AC133+ culture becomes heterogeneous population, containing highly proliferative, more immature cells and less immature cells with more pronounced endothelial characteristics. However, the answer to this question cannot be provided by the current study and will necessitate analysis at the single cell level, which is part of our future studies.

One of the pivotal properties of EPCs is the ability to give rise to the functional progeny, i.e. mature endothelial cells. Considering that our findings demonstrated that at mid time point in the culture cell population became heterogeneous, it was critically important to determine whether ability to differentiate and carry the function of endothelial cells was affected by long term *in vitro* expansion. The main features indicative of acquiring endothelial phenotype are changes in cellular morphology and expression of specific mature endothelial markers. Many groups considered the expression of CD31, VEGF receptor 2 (KDR) and von Willebrand factor as the expression hallmarks of endothelial cell type. In addition to these markers, we demonstrated that long term expanded CB AC133+ cultured under differentiation conditions also expressed ICAM-1 (CD54), E-selectin (CD62E) and SLAM (CD150). Cells maintained under the growth (non-differentiating) conditions also expressed CD105, CD29 and CD184; however, these molecules were strongly upregulated and expressed on 100% of cells that were cultured under the differentiating conditions. The CD105 increase under the differentiating conditions also correlated to the data gained from the study by Senegaglia et al [38]. Collectively, these data are in agreement with the expected changes in cell shape, motility and ability to migrate that are associated with EC differentiation and at the same time are the properties necessary to carry out angiogenic effects of mature endothelial cells. Altogether, we show that under the appropriate microenvironmental cues CB AC133+ were able to give rise to the cells with endothelial characteristics with no difference between the cells expanded for 10–15 days and the one expanded for 20–25 days.

Functional testing using matrigel tube formation assay showed that CB AC133+ failed to form cord like structures when plated alone. The observed results contrasted the previous reports on CB AC133+ cells [38] that indicated self-sufficiency of these cells for generating tube like structures. However, our results from CB AC133+ cells – HDMVECs co-culture matrigel experiments showed that CB AC133+ cells migrated and co-localized with the HDMVECs that formed tube like structures. In addition, HDMVECs incubated in the presence of EPCs' supernatants (w/o EPCs) formed complete tubes in matrigel. Diversity of CB AC133+ cells' growth culture conditions used by various groups may explain the contrary results reported in the literature that concerns particular aspects of progenitor function and it is possible that certain growth factors would promote or inhibit some of the functional properties. In particular, growth conditions used by most of the researchers that promoted cell attachment in the presence of FBS may have influenced the formation of more mature cells that were able to make tubes by themselves. On the other hand, our data indicate that EPCs may exert their proangiogenic effect in a paracrine fashion, as previously indicated by findings showing that under ischemic conditions EPC may induce neovascularization by secreting growth factors and cytokines (such as VEGF) that induce sprouting angiogenesis by the neighboring endothelium [44].

One of the hallmarks of tissue ischemia is increase in the SDF-1 expression, generating strong migratory signal for circulating EPCs [45], [46]. In addition to responsiveness to ischemic stimuli, functional EPCs are characterized by the ability to respond to inflammatory stimuli (such as RANTES) as well. The capacity to migrate in response to the such stimuli is crucial for endothelial progenitors to exert their angiogenic potential and participate in neovascularization processes and the importance of this chemotaxis was demonstrated by many groups [47], [48], [49]. Here we also showed that that during the long term culturing, CB AC133+ progenitors maintained functional characteristics with regard to chemotaxis and exhibited similar migratory response to SDF-1α and RANTES at day 10–15 and at day 25–30 of culture. In addition, we demonstrated that long term *in vitro* expansion did not impede the ability to migrate and incorporate into neovascularization sites under *in vivo* conditions. Our previous studies on tumor animal models also showed that locally [50] and systemically [51] administered FePro labeled CB AC133+ cells migrated and incorporated into the tumor vasculatures.

In summary, our results indicate that long term culturing can give significant quantities of CB AC133+ cells that preserved the capacity to differentiate into functional mature ECs and can be used in the studies relevant to the development of future angiogenic therapies as well as in studies on progenitor cell biology and development.
